# The Role of Energy-Dispersive X-Ray Fluorescence to Predict Mineral Content in Untreated Bovine Plasma

**DOI:** 10.3390/ani15081133

**Published:** 2025-04-14

**Authors:** Davide Martini, Silvia Magro, Marta Pozza, Mauro Penasa, Massimo De Marchi

**Affiliations:** Department of Agronomy, Food, Natural Resources, Animals and Environment, University of Padova, Viale dell’Università 16, 35020 Legnaro, PD, Italy; davide.martini.5@phd.unipd.it (D.M.); marta.pozza.2@unipd.it (M.P.); mauro.penasa@unipd.it (M.P.); massimo.demarchi@unipd.it (M.D.M.)

**Keywords:** cattle, diagnostic kit, health, non-destructive approach, reference analysis

## Abstract

Minerals and trace elements are vital for numerous physiological processes in mammals. The current reference analysis for mineral determination in biological matrices is inductively coupled plasma mass spectrometry (ICP-MS). This analysis is costly, time-consuming, and destructive. While commercial kits are a viable alternative to ICP-MS due to the lower cost, their limit lies in the ability of determining only one mineral at a time. Energy-dispersive X-ray fluorescence (ED-XRF) has been proposed as a potential alternative for the rapid determination of mineral concentration in biological matrices. This study evaluated the accuracy of ED-XRF as an alternative to commercial diagnostic kits to predict the concentrations of sodium, magnesium, phosphorus, chloride, potassium, calcium, iron, and selenium in cattle plasma without sample pretreatment, potentially reducing the time of analysis compared to commercial kits and costs and labor compared to ICP-MS. Reference mineral concentrations were determined in 277 samples using in vitro diagnostics regulation-certified commercial diagnostic kits. The results indicated a moderate prediction accuracy only for potassium. For the other minerals, the prediction accuracy of ED-XRF was insufficient, which suggests that some degree of sample preparation is necessary to improve the determination of minerals in plasma.

## Simple Summary

Minerals are essential for animal health but measuring their levels in blood with traditional methods is costly and time-consuming. While some studies have explored the use of energy-dispersive X-ray fluorescence in biological matrices, including plasma, none has assessed its applicability in untreated samples. This study evaluates the potential of energy-dispersive X-ray fluorescence to predict the concentrations of sodium, magnesium, phosphorus, chloride, potassium, calcium, selenium, and iron in unprocessed plasma samples.

## 1. Introduction

Inorganic elements, such as minerals and trace elements, play a crucial role in physiological processes in mammals, including cattle [1]. Maintaining a proper balance of these elements is essential, as both deficiencies and excesses lead to disorders affecting metabolic functions, immune response, and overall productivity [2]. Therefore, monitoring mineral levels allows for the early identification of imbalances, enabling timely dietary adjustments and supplementation strategies. There are several methods to assess the mineral concentration in whole blood and serum, such as inductively coupled plasma optical emission spectroscopy, inductively coupled plasma mass spectrometry (ICP-MS) [3], and commercial diagnostic kits based on ion-selective electrode analysis (ISE), colorimetry, and ultraviolet (UV) complexometry. Each of these approaches, however, has limitations. ICP-MS is time-consuming, costly, destructive, and requires sample preparation, such as matrix decomposition [4]. Similarly, ISE requires a specific electrode for each mineral, limiting the analysis to one mineral at a time. Colorimetric methods demand significant time for accurate processing [5]. As for UV complexometry, results’ interpretation can be challenging due to the overlap of absorption bands and the necessity for theoretical calculations [6].

In this context, there is significant interest in utilizing energy-dispersed X-ray fluorescence (ED-XRF) to detect mineral concentration as it requires little to no preparation and allows for the simultaneous analysis of multiple minerals and 10 to 15 samples per hour, depending on the matrix. Previous studies suggested that ED-XRF can be applied to various untreated matrices that are either solid or liquid, such as milk and cheese. Pozza et al. [7] reported a coefficient of determination (R^2^) of >0.65 for magnesium (Mg), phosphorus (P), potassium (K), and calcium (Ca) in non-lyophilized milk and skim milk powder. Visentin et al. [8] obtained R^2^ > 0.35 for P and K in non-lyophilized milk and > 0.40 for P, S, K, and Ca in cheese. For other minerals, the R^2^ was close to zero. To our knowledge, only a few studies have tested ED-XRF on serum or plasma, with a specific focus on humans. However, the protocols required sample preparation, such as lyophilization or dehydration, which are time-consuming [9,10,11,12]. Therefore, the present study aimed to evaluate whether ED-XRF can accurately predict the concentration of various minerals, including sodium (Na), Mg, P, chloride (Cl), K, Ca, iron (Fe), and selenium (Se), in bovine plasma without any sample pretreatment.

## 2. Materials and Methods

### 2.1. Plasma Samples

A total of 277 bovine plasma samples were obtained from previous studies [13,14]. Briefly, 231 samples were collected from Charolais young bulls during a feeding trial [14] and 46 samples from early-lactation dairy cows (days in milk < 38) [13]. The blood samples were taken from the external jugular vein in bulls and the medial caudal vein in cows, following the same standard protocol and using 9 mL Vacuette^®^ LH—lithium heparin—blood collection tubes (Greiner Bio-One GmbH, Kremsmünster, Austria). The tubes were then placed into a box refrigerated at +4 °C until the end of the sampling session. The blood tubes were centrifuged at 1500× *g* for 15 min at 4 °C within 2 h of collection. The plasma was then aliquoted and stored at −80 °C until analysis. An aliquot of each plasma sample was transported to two laboratories: the Istituto Zooprofilattico Sperimentale delle Venezie (Legnaro, Italy) and EcamRicert (Malo, Italy). The determination of Na, Mg, P, Cl, K, Ca (mmol/L), and Fe (µg/L) was conducted at the Istituto Zooprofilattico Sperimentale delle Venezie using in vitro diagnostics regulation (IVDR) certified commercial diagnostic kits (Roche Diagnostics, Basilea, Switzerland). The kits, which served as reference methods for all mineral elements except Se, employed ISE for the measurement of Na, Cl, and K, colorimetric techniques for Mg, Ca, and Fe, and UV complexometric methods for P. The performance of the kits is reported in Table 1.

**Figure 1:**
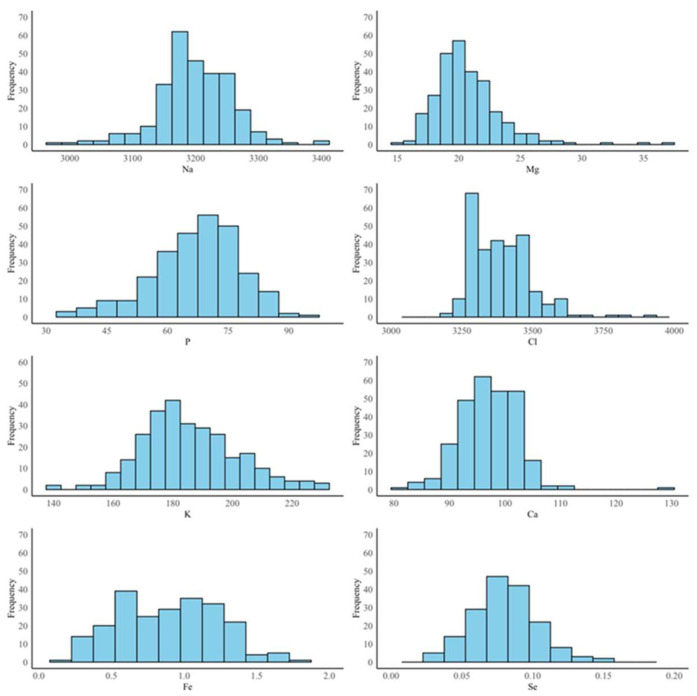
Distribution of minerals in bovine plasma (mg/kg) determined through commercial diagnostic kits for sodium (Na), magnesium (Mg), phosphorus (P), chloride (Cl), potassium (K), calcium (Ca) (*n* = 277), and iron (Fe) (*n* = 228), and inductively coupled plasma optical emission spectroscopy for selenium (Se) (*n* = 174).

With regard to Se (µg/dL), this was determined at EcamRicert using ICP-MS. Fe and Se were measured only in bull plasma [14] because the trial on dairy cows did not include the collection of information on these minerals [13]. A total of 3 samples had values below the limit of detection for Fe and 57 samples for Se. The mineral element concentrations were transformed to mg/kg. For Na, Mg, P, Cl, K, and Ca, the transformation was conducted using the molecular weight (g/mol) of the individual mineral (Na = 22.99, Mg = 24.31, P = 30.97, Cl = 35.35, K = 39.10, and Ca = 40.08), and the specific weight of the plasma was assumed to be equivalent to that of water. Regarding Fe and Se, the values were converted dividing by 0.001 and 0.01, respectively. Overall, the minerals in the plasma (mg/kg) were normally distributed (Figure 1).

### 2.2. ED-XRF Analysis

**Figure 2:**
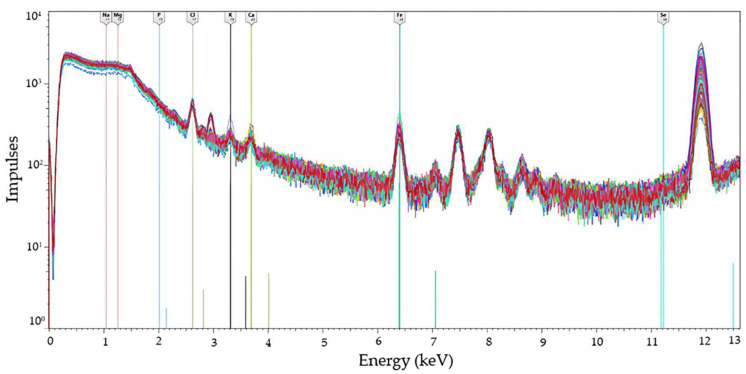
X-ray fluorescence spectrum of untreated bovine plasma samples (*n* = 277, except for Fe with *n* = 228 and Se with *n* = 174) and excitation energies associated with mineral elements.

An aliquot of untreated plasma samples (i.e., samples which did not undergo any further preparation after the centrifugation) was analyzed in the laboratory of the Department of Agronomy, Food, Natural resources, Animals and Environment of the University of Padova (Legnaro, Italy) using Spectro Xepos 5P ED-XRF (Ametek, Kleve, Germany) equipped with an X-ray tube anode of Lead and Cobalt (65–35), 50 Kev voltage, and 2 mA current. Mineral quantification was based on Na k-α (1.04 Kev), Mg k-α (1.25 Kev), P k-α (2.01 Kev), Cl k-α (2.82 Kev), K k-α (3.31 Kev), Ca k-α (3.69 Kev), Fe k-α (6.40 Kev), and Se k-α (11.22 Kev). The potential for signal overlap was addressed through the method of influence coefficients [15]. This was implemented and applied through the ED-XRF software (v. 3.9.3) as a correction factor for matrix effects and their quantification. Before spectral acquisition, the instrument was calibrated according to the manufacturer’s instructions. Each sample was placed in ED-XRF plastic cups (32 mm diameter and 24 mm height; Ametek, Kleve, Germany) and before the beginning of the analysis, matrix type and weight were provided to the instrument to enhance the precision of the ED-XRF, which depends on the weight and texture of the material that the X-rays must traverse. The chamber of the ED-XRF instrument was filled with helium to reduce the attenuation of low-energy X-rays. Helium, being less dense than air, minimized scattering and absorption, particularly for lighter minerals, thereby improving the detection sensitivity for these mineral elements. The instrument required 4 min to analyze each sample. The resulting spectra for each sample are depicted in Figure 2.

### 2.3. Statistical Analysis

The energy-dispersive X-ray fluorescence software (v. 3.9.3) reported raw data as energy (Kev) versus normalized impulse (counts per second) and converted normalized impulses to the predicted concentration of the *i*th element (Na, Mg, P, Cl, K, Ca, Fe, Se) using the following formula, based on fundamental parameters’ quantification:

(1) $$C_{i}=K_{0}+K_{1}*I_{i}*\mu$$

where *C_i_* is the predicted concentration of the *i*th element; *K*_0_ is the offset of the calibration; *K*_1_ is the slope of the calibration; *I_i_* is the fluorescence intensity of the *i*th element; and *µ* is the mass attenuation coefficient, calculated as the sum of individual influences between the *i*th element and other interfering elements. The ED-XRF calibration was performed on the entire dataset as a regression between the concentrations of the *i*th mineral element (mg/kg) measured by the commercial diagnostic kits (Na, Mg, P, Cl, K, Ca, Fe) or ICP-MS (Se) as reference against the predicted value (mg/kg) from the ED-XRF, using the fundamental parameters’ formula, as previously described. The same regression was iterated for each mineral element included in this study, leading to the development of 8 calibration equations. Before final calibration, the dataset was checked for outliers. For this purpose, the difference between the reference value from commercial diagnostic kits (Na, Mg, P, Cl, K, Ca, Fe) or ICP-MS (Se) and the predicted value from ED-XRF was calculated [16,17], and values deviating more than 3 standard deviations from the respective mean (6 outliers for Na, 2 for Mg, 6 for P, 9 for Cl, 6 for K, 5 for Ca, 3 for Fe, and 2 for Se) were discarded. Following the removal of the outliers, each regression equation was recalculated. The agreement between reference and predicted values was evaluated through R^2^.

## 3. Results and Discussion

### 3.1. Descriptive Statistics

Descriptive statistics of the minerals determined through reference analyses are reported in Table 2. Cl and Na were the most abundant in bovine plasma, with average concentrations of 3390.21 and 3195.36 mg/kg, respectively. On the other hand, Fe and Se were the least abundant, with average concentrations of 0.90 and 0.09 mg/kg, respectively. The average concentrations of other minerals were 185.76 mg/kg for K, 97.05 mg/kg for Ca, 66.93 mg/kg for P, and 20.71 mg/kg for Mg. The concentrations of Na, Mg, P, Cl, K, and Ca were in line with those reported by McAdam and O’Dell [18], who investigated these minerals in the plasma of 20 lactating Holsteins. Luna et al. [19] conducted a study on 20 Holstein cows of parity 2 to 3 and from 60 to 90 days in milk, reporting slightly lower mean concentrations of Mg (36.00 mg/kg) and Ca (108 mg/kg) and an approximately four times greater mean concentration of P (255.00 mg/kg) compared to the present study. Herdt et al. [20] reported similar concentrations of Mg and Ca in a sample of 121 herds compared to our study. Conversely, the concentrations of Fe and Se in the present study were slightly lower than those reported by Herdt and Hoff [21] and Mehdi and Dufrasne [22]. In particular, Herdt and Hoff [21] found Fe and Se concentrations which ranged from 1.10 to 2.50 ug/mL (equivalent to mg/kg) and 65.00 to 140.00 ng/mL (i.e., 0.065 to 0.14 mg/kg), respectively, in 1585 adult cattle distributed across 165 herds. A similar range for Se (0.08 to 0.16 mg/kg) was reported in cattle in a review of Mehdi and Dufrasne [22].

### 3.2. Comparison Between Reference Methods and ED-XRF

**Figure 3:**
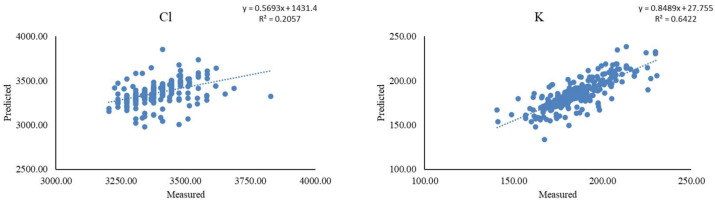
Plot of measured (through commercial diagnostic kits) versus predicted (through energy-dispersive X-ray fluorescence) concentration (mg/kg) of minerals (chloride, Cl, and potassium, K) with coefficient of determination (R^2^) > 0.10.

After removal of the outliers, the comparison between the reference values from diagnostic kits (Na, Mg, P, Cl, K, Ca, and Fe) or ICP-MS (Se) and the predicted values from ED-XRF highlighted a moderate accuracy for K (R^2^ = 0.64), followed by Cl (R^2^ = 0.21). The other minerals had very low R^2^ (<0.10), which indicates the absence of a relationship between the reference and the predicted values. For clarity and relevance, only scatter plots of measured versus predicted concentrations of K and Cl and the respective calibration equations are displayed in Figure 3. The ability of ED-XRF to predict K better than the other minerals could be attributed to its relatively large molecular weight and its sufficiently high concentration in plasma. On the other hand, Cl had the greatest mean concentration among the minerals and presented a molecular weight similar to that of K. The low correlation between the reference and predicted values could have been due to Cl being more susceptible to interferences in the matrix, such as water.

Custódio et al. [12] demonstrated that ED-XRF is a reliable technique to determine K, Ca, Fe, Cu, Zn, Se, Br, Rb, and Pb in blood samples that have been lyophilized and pressed into pellets. Lyophilization reduces the water content and thus decreases the background noise from water, while compressing the sample into pellets minimizes matrix attenuation. This process allows X-rays to penetrate the full sample thickness, enhancing the accuracy and consistency of the fluoresced X-ray measurements by reducing bias caused by sample variability [23]. The predictions of Custódio et al. [12] were consistent with the values obtained from certified reference materials, falling within the established confidence intervals. The comparison of our findings with those of Pozza et al. [7] and Visentin et al. [8] highlighted that plasma analysis may exhibit heightened susceptibility to interferences, including background noise from water content, which can adversely affect the accuracy of results. Pozza et al. [7], who used the same experimental setup as the present study, reported R^2^ of 0.65 for Mg, 0.95 for P, 0.99 for S, 0.80 for K, and 0.98 for Ca in skim milk and whey powders during external validation. Visentin et al. [8], who adopted the same ED-XRF instrument but using the Lucas–Tooth algorithm instead of the fundamental parameter approach, obtained R^2^ of 0.39 for Ca in non-lyophilized milk and 0.60 in cheese. Notably, in the studies of Pozza et al. [7] and Visentin et al. [8], K was among the best predicted minerals, reflecting its less susceptibility to interferences than other minerals.

Minerals with a relatively low molecular weight, such as Na, Mg, and P, tended to have lower excitation energies, which made them more prone to interference in the spectral region, as depicted in Figure 2. In contrast, minerals with greater molecular weight such as Fe had greater excitation energies, which typically resulted in a more distinct peak in the spectrum (Figure 2). However, the poor performance observed for Fe in this study could be attributed to its low concentration in the plasma, which was close to the detection limit of the instrument. The limit of detection could also explain the inability of the instrument to quantify Se, which was the lowest concentrated mineral in plasma. Despite being the heaviest mineral, it had high background noise, similarly to lighter minerals. The poor prediction of Cl, K, and Ca may have resulted from various interferences within the matrix. These interferences could have arisen from overlapping spectral lines, matrix effects, or other minerals present in the sample that affected the accuracy of detection and quantification. In addition, other compounds in the matrix, such as proteins, could have interfered and contributed to reduce the signal-to-noise ratio. Another potential cause for the poor prediction accuracy could be attributed to the utilization of commercial diagnostic kits, which, despite being IVDR-certified, do not represent the gold standard for mineral analysis in blood samples.

## 4. Conclusions

ED-XRF failed to provide accurate predictions for the minerals considered in this study, except for a moderate capability in predicting K. When applied to untreated samples, ED-XRF is not an adequate method for the comprehensive mineral analysis of cattle plasma. Future research should focus on optimizing sample preparation techniques such as dehydration, which can concentrate mineral content and reduce matrix effects, or pelletization, which enhances sample uniformity and minimizes surface irregularities, thereby improving measurement accuracy. Additionally, further comparative studies with other matrices are recommended to explore the full potential and limitations of ED-XRF for mineral analysis in biological samples.

## Figures and Tables

**Table 1:** Analytical techniques ^1^ used in the assay kits for the measurement of minerals, along with their sensitivity and intra-assay/inter-assay coefficient of variation (CV).

Mineral ^2^	Analytical Technique	Sensitivity (mmol/L)	Intra-Assay CV (%)	Inter-Assay CV (%)
Na	ISE	80	0.3	0.5
Mg	Colorimetric (xylidine Blue)	0.1	1.1	1.3
P	UV Complexometric (phosphomolybdate without reduction)	0.1	0.7	1.4
K	ISE	1.5	0.5	0.7
Cl	ISE	60	0.3	0.6
Ca	Colorimetric (o-Cresolphthalein)	0.2	1.0	1.6
Fe	Colorimetric (ferrozine without deproteinization)	0.9	1.3	1.8

^1^ ISE = ion-selective electrode; UV = ultraviolet. ^2^ Na = sodium; Mg = magnesium; P = phosphorus; K = potassium; Cl = chloride; Ca = calcium; and Fe = iron.

**Table 2:** Descriptive statistics ^1^ of reference values for minerals concentration (mg/kg) in plasma determined by commercial diagnostic kits for Na, Mg, P, Cl, K, Ca, and Fe, and inductively coupled plasma mass spectrometry for Se, alongside fitting statistics of the prediction models ^2^.

Mineral ^3^	Reference				Reference vs. ED-XRF ^4^	
	Mean	SD	CV, %	Range	R^2^	SE
Na	3195.36	56.06	1.75	2965.71–3402.71	0.01	56.33
Mg	20.71	2.71	13.09	15.07–37.19	0.00	2.72
P	66.93	11.61	17.35	32.21–93.53	0.01	10.75
Cl	3390.21	102.72	3.03	3203.85–3934.95	0.21	83.97
K	185.76	19.39	10.44	140.76–230.69	0.64	9.52
Ca	97.05	5.29	5.45	81.76–128.65	0.06	5.13
Fe	0.90	0.47	52.23	0.06–1.90	0.03	0.35
Se	0.09	0.07	80.12	0.03–0.15	0.09	0.02

^1^ SD = standard deviation; and CV = coefficient of variation. ^2^ R^2^ = coefficient of determination; and SE = standard error. ^3^ Na = sodium; Mg = magnesium; P = phosphorus; Cl = chloride; K = potassium; Ca = calcium; Fe = iron; and Se = selenium. ^4^ ED-XRF = energy-dispersed X-ray fluorescence.

## Data Availability

None of the data were deposited in an official repository. The data that support this study are available from the corresponding author upon reasonable request.

## Abbreviations

The following abbreviations are used in this manuscript:

ICP-MS	Inductively Coupled Plasma Mass Spectrometry
ED-XRF	Energy-Dispersed X-ray Fluorescence
Na	Sodium
Mg	Magnesium
P	Phosphorus
S	Sulfur
Cl	Chloride
K	Potassium
Ca	Calcium
Fe	Iron
Se	Selenium
ISE	Ion-Selective Electrode
UV	Ultraviolet
R2	Coefficient of Determination
IVDR	In Vitro Diagnostics Regulation
CV	Coefficient of Variation
